# The Use of AI in Diagnosing Diseases and Providing Management Plans: A Consultation on Cardiovascular Disorders With ChatGPT

**DOI:** 10.7759/cureus.43106

**Published:** 2023-08-07

**Authors:** Ayesha Rizwan, Tahira Sadiq

**Affiliations:** 1 Medicine and Surgery, Islamic International Medical College, Islamabad, PAK; 2 Community Medicine, Islamic International Medical College, Islamabad, PAK

**Keywords:** ai consultation, cardiovascular disorders, diagnosis and management, artificial intelligence chatgpt-4, ai and robotics in healthcare

## Abstract

Background: Cardiovascular diseases (CVDs) have remained the leading causes of death worldwide and substantially contribute to loss of health and excess health system costs. According to WHO, cardiovascular diseases (CVDs) take an estimated 17.9 million lives each year. One of the reasons for an immensely high fatality in CVDs is lack of efficient diagnosis and prompt treatment. Timely recognition and management are crucial to minimize mortality. In the advancing world, AI (artificial intelligence) and machine learning technologies continue to progress, this advancement has opened new avenues for innovative approaches in the field of medicine. Despite the rapid development in the field of AI, there is a limited understanding of the potential benefits among clinicians and medical practitioners.

Methods: In this study, we aimed to investigate the potential that the AI language model holds to assist health practitioners in the diagnosis and treatment of cardiovascular disorders. We asked Chat Generative Pre-trained Transformer (ChatGPT) 10 hypothetical questions simulating clinical consultation. The responses given by ChatGPT were accessed for its accuracy and accessibility by a team of medical specialists and cardiologists with extensive experience in managing cardiovascular disorders.

Result: Out of the 10 clinical scenarios inserted in ChatGPT, eight were perfectly diagnosed, however, the other two answers given by ChatGPT were not entirely incorrect since those conditions were associated with the actual diagnosis. Furthermore, the management plans and the treatment protocols that were given by ChatGPT were in line with the literature and current medical knowledge. The exact drug names and regimens were not provided but a general guideline that was given by this AI tool is definitely beneficial for junior doctors in getting an idea on how to proceed or refresh their previous knowledge.

Conclusion: ChatGPT is a valuable resource in the field of medicine. Its comprehensive and properly organized response in an understandable language has made it an effective and efficient tool to be used. However, it is crucial to note that its limitations, such as the need for all associated and typical signs, symptoms, and physical examination findings, and its inability to personalize treatments need to be acknowledged.

## Introduction

Cardiovascular diseases (CVDs) have remained the leading causes of death worldwide and substantially contribute to loss of health and excess health system costs. Deaths from cardiovascular disease surged 60% globally over the last 30 years. According to a recent report from the World Heart Federation on 20 May 2023, deaths from cardiovascular disease (CVD) jumped globally from 12.1 million in 1990 to 20.5 million in 2021 [1]. According to WHO, CVDs take an estimated 17.9 million lives each year. One of the reasons for an immensely high fatality in CVDs is the lack of efficient diagnosis and prompt treatment [2]. Timely recognition and management are crucial to minimize mortality.

In the advancing world, artificial intelligence (AI) and machine learning technologies continue to progress, this advancement has opened new avenues for innovative approaches in the field of medicine. Despite the rapid development in the field of AI, there is a limited understanding of the potential benefits for clinicians and medical practitioners. AI language-generated tools such as Chat Generative Pre-trained Transformer (ChatGPT) possess remarkable capabilities to disseminate medical information [3]. This tool does not only generate human-like text but the answers provided are extracted from multiple sites and research papers comprehensively. ChatGPT has proven to be highly beneficial in writing literature reviews, essays, and research papers, and providing answers to academic questions, however, its potential in the field of medicine, medical consultation, and patient care is yet to be discovered [4]. To explore this potential, the authors have conducted a simulated cardiology consultation using ChatGPT to generate answers to 10 cardiology-based clinical scenarios, subsequently evaluating its response.

## Materials and methods

In this study, we aimed to investigate the potential that the AI language model holds to assist health practitioners in the diagnosis and treatment of cardiovascular disorders. For this purpose ChatGPT, which is one of the largest language models currently accessible to the public, was used and its capacity to provide medical consultation, accuracy, and effectiveness was evaluated.

Study design

We asked ChatGPT 10 hypothetical questions simulating clinical consultation. The questions were clinical scenarios typical of each of the 10 cardiovascular disorders consisting of the signs and symptoms commonly seen in clinical settings coherent with the research and literature present. The scenario objective was to cover a broad cross-section of information that is needed by caregivers in the field of medicine. The responses given by ChatGPT were accessed for its accuracy and accessibility by a team of medical specialists and cardiologists with extensive experience in managing cardiovascular disorders. By evaluating the responses provided we aimed to establish the authenticity of AI-generated models such as ChatGPT in simulating clinical consultation in the field of medicine.

Inclusion and exclusion criteria

ChatGPT employs a probabilistic algorithm generating a diverse array of responses. ChatGPT4 was chosen to be used in our research as it is a version widely available and freely accessible to the public. The initial responses provided were documented refraining from any further clarification or correction; furthermore, meticulous attention was given to ensure that all the scenarios inserted were free from grammatical or syntactical errors. All of the scenarios were inserted on the same day by one of the authors to ensure that it is devoid of any variation. Alternative chatbots were not considered for this study as they demonstrated inferior capabilities when compared to the version used.

The primary objective of this study was to assess the performance of ChatGPT4 as a representative of advanced AI-generated models within the specific context of consultation related to cardiovascular disorders.

## Results

**Figure 1:**
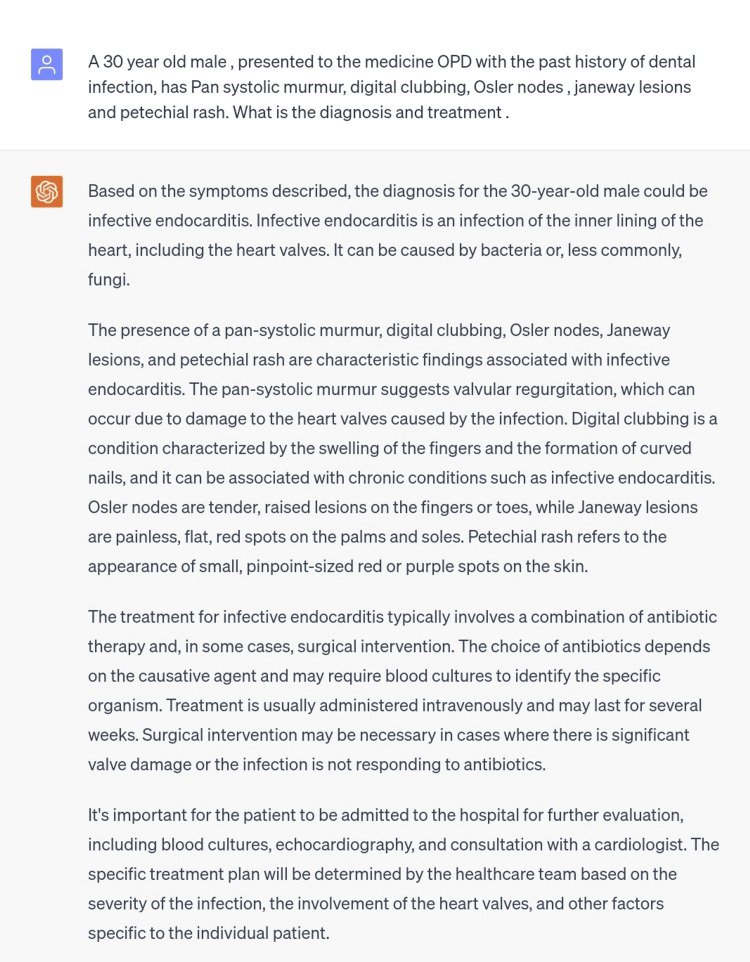
Scenario of infective endocarditis.

The first scenario generated was of infective endocarditis, which has an incidence rate of 3-10 cases in every 100,000 people. This condition is caused by bacterial infections commonly seen in IV drug abusers, dental infections, or patients with prosthetics [5]. The typical signs and symptoms of this condition such as pan systolic murmur Osler nodes, Janeway lesions, digital clubbing, and petechial rash were mentioned in the scenario typed in ChatGPT. The diagnosis made by ChatGPT was correct and the treatment plan provided was also coherent with the treatment guidelines, however, it is to note that the treatment as a whole, was general and can act as a guide in planning out a precise and comprehensive treatment plan. Rather than mentioning the names of drugs or the names of surgical interventions, a general treatment regimen in an appropriate sequence was given. Therefore, it has been observed that in the case scenario of infective endocarditis, the diagnosis made was correct and the treatment plan can be used for academic purposes rather than being dependent on clinical, however, it can assist junior doctors in mapping out the treatment plan (Figure 1).

**Figure 2:**
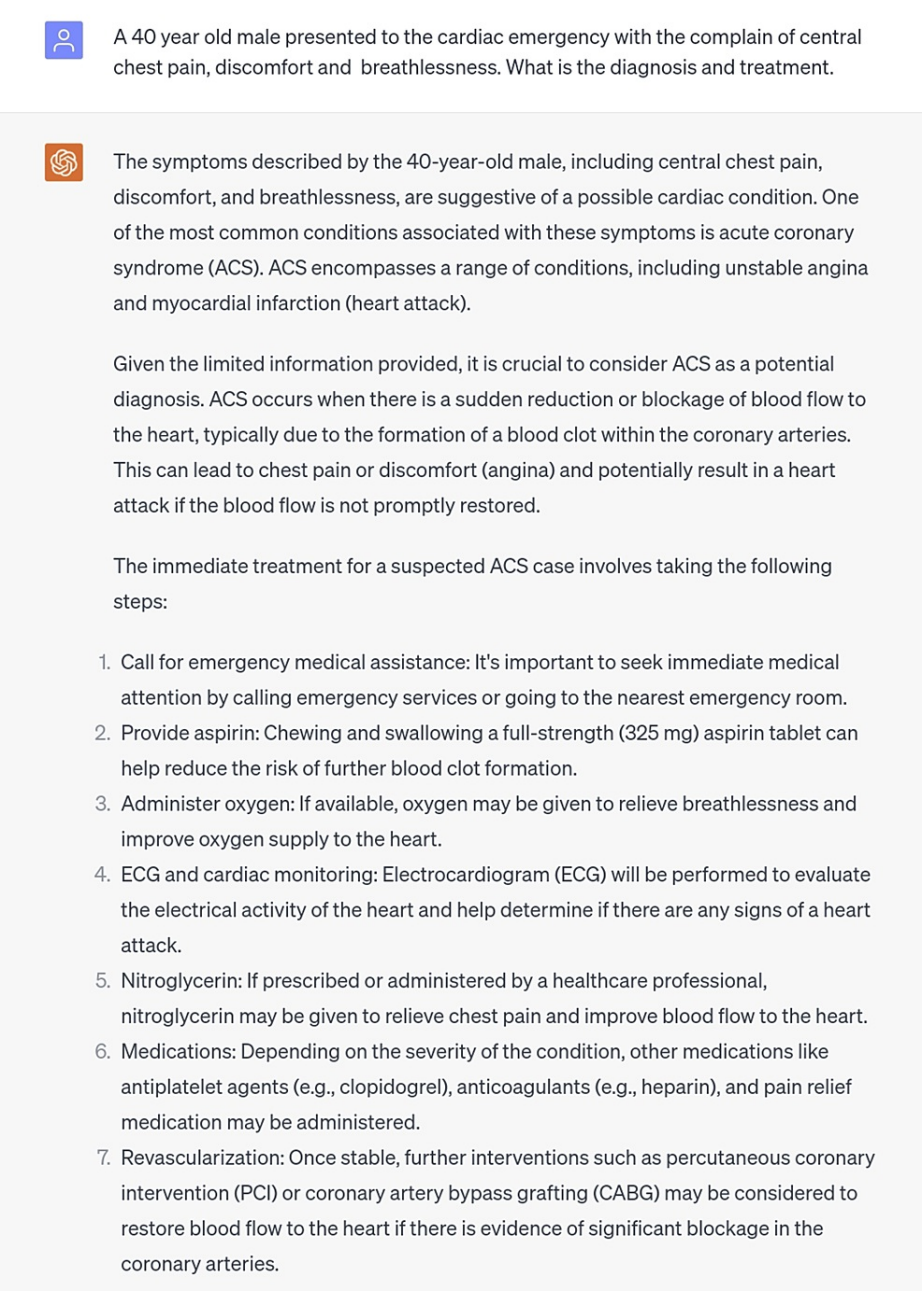
Scenario of angina pectoris.

The second scenario was of angina pectoris which is a complex symptom caused by transient myocardial ischemia [6]. The answer provided by ChatGPT was acute coronary syndrome. In this particular scenario, it is to note that acute coronary syndrome (ACS) is one of the common causes that present with stable angina. This has led us to the conclusion that a subset of symptoms is not sufficient rather ECG findings are also needed in order to create a definite diagnosis. This is not a limitation but a common situation that is commonly experienced by medical practitioners; furthermore, the need for ECG was also mentioned in the answer given by ChatGPT and the management protocol is also satisfactory (Figure 2).

**Figure 3:**
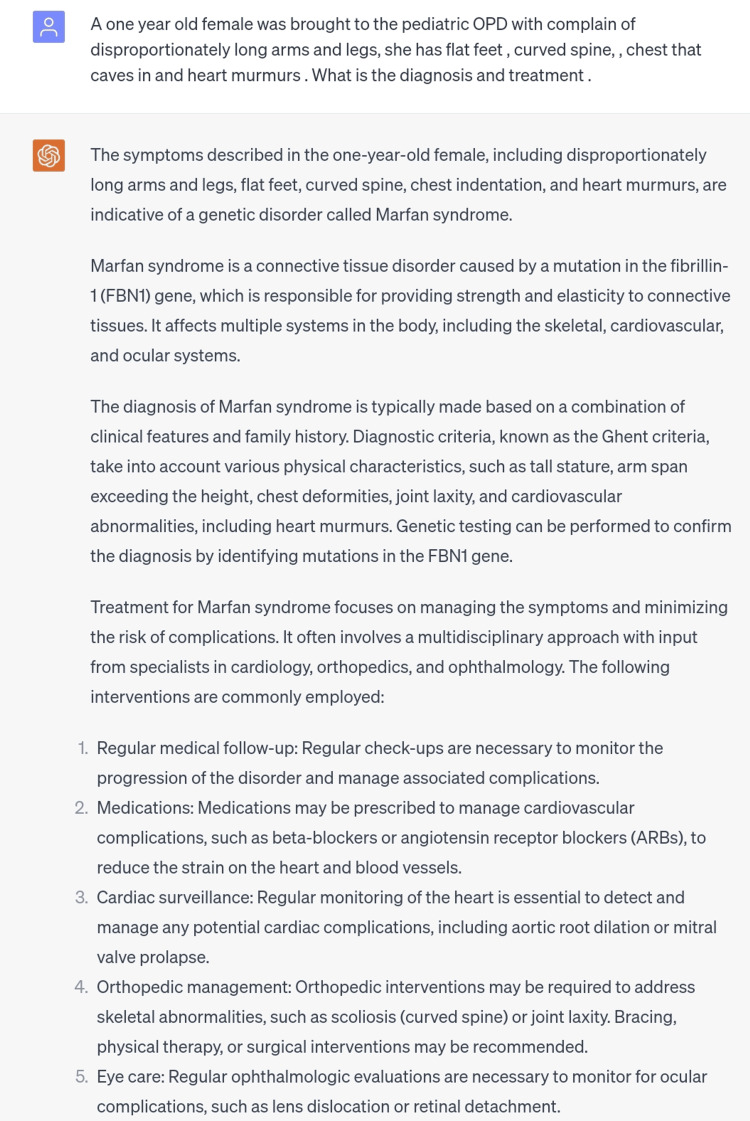
Scenario of Marfan syndrome.

The third scenario was of Marfan syndrome which is caused by a mutation in a gene called FBN1 [7]. The clinical picture of this condition was inserted in ChatGPT and the response given consisted of the correct diagnosis and an appropriate treatment plan which again, was general. Rather than mentioning the exact treatment regimen a general guideline was provided (Figure 3).

**Figure 4:**
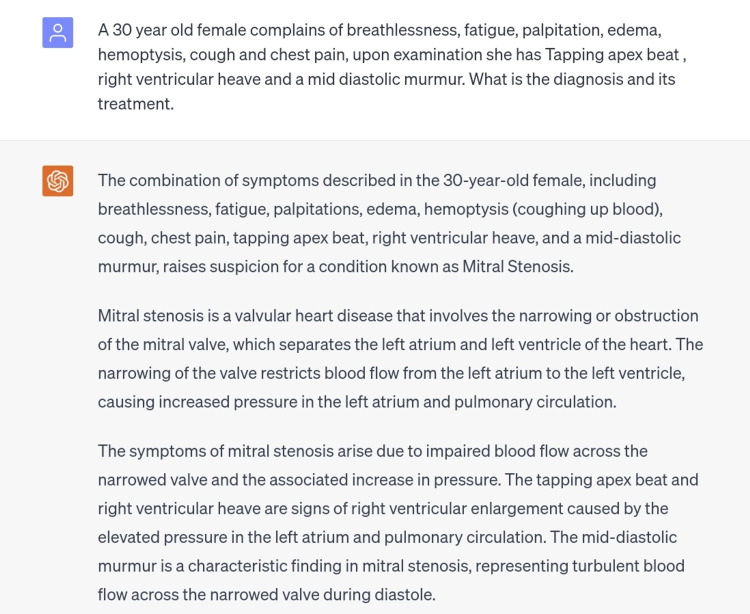
Scenario of mitral stenosis with the findings of CVS examination. CVS: cardiovascular system

**Figure 5:**
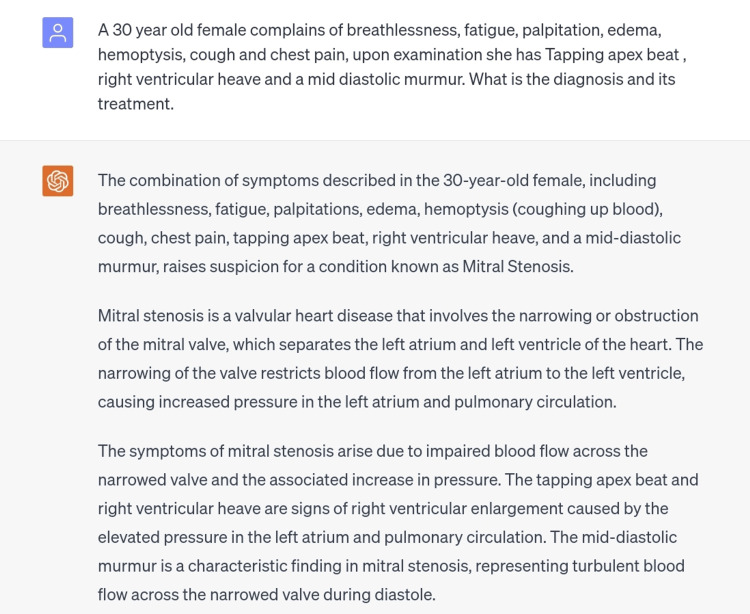
Similar scenario but without the CVS examination findings. CVS: cardiovascular system

The fourth scenario was mitral stenosis. Since this condition requires signs observed on detailed cardiovascular system (CVS) physical examination as well as the symptoms, a comprehensive scenario was typed in the chatbot which gave the correct diagnosis (Figure 4) [8]. An important thing to note here was that when the same scenario was typed in without the details of the CVS examination the diagnosis made was incorrect, pulmonary hypertension was the diagnosis made which is a condition commonly associated with mitral stenosis (Figure 5).

**Figure 6:**
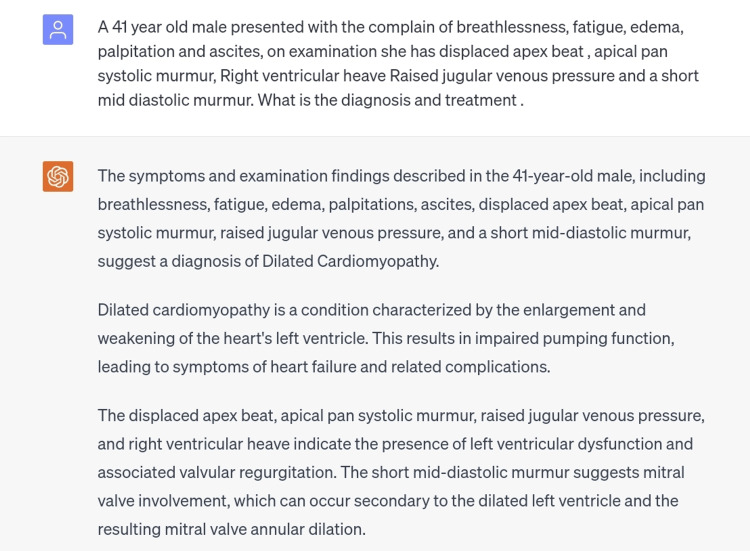
Scenario of mitral regurgitation.

The fifth scenario was mitral regurgitation, which was typed in the chatbot with all its associated signs and symptoms along with the CVS examination findings, however, the diagnosis made by ChatGPT was dilated cardiomyopathy [9]. Mitral regurgitation is a frequent comorbid condition with dilated cardiomyopathy (DCM) as it deteriorates left ventricular volume and ejection fraction (Figure 6).

**Figure 7:**
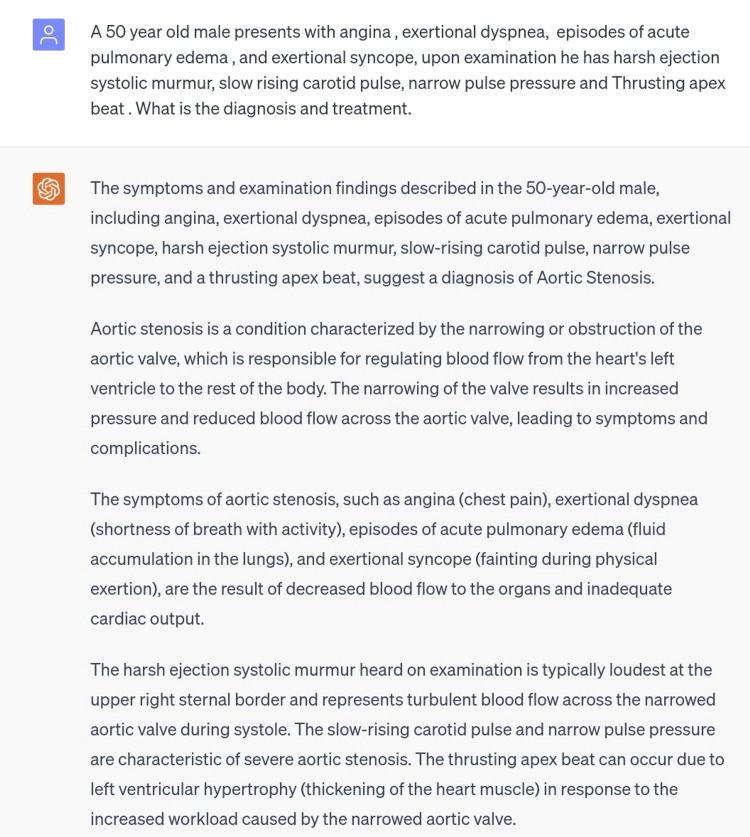
Scenario of aortic stenosis.

**Figure 8:**
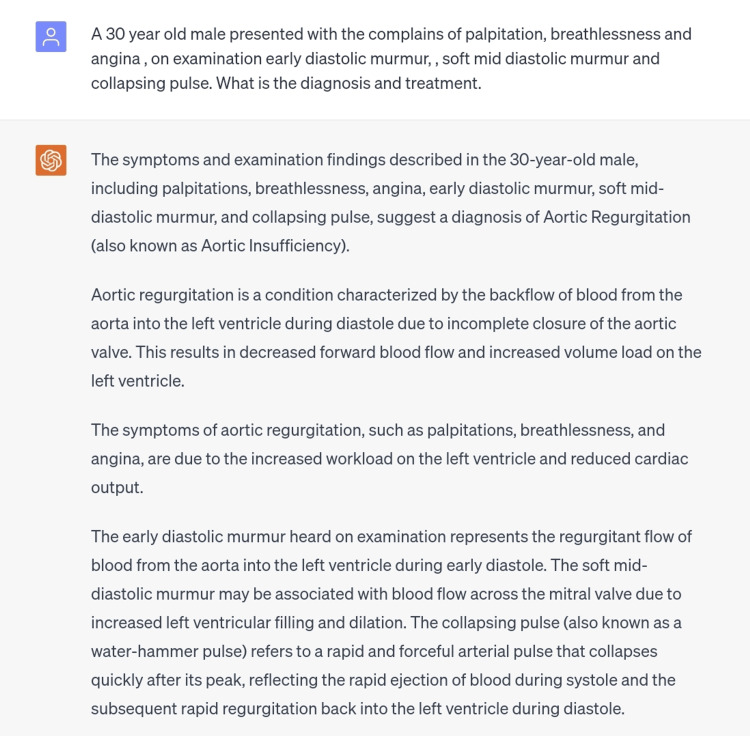
Scenario of aortic regurgitation.

The next sixth and seventh scenarios were of aortic stenosis and aortic regurgitation, respectively (Figures 7, 8). For both conditions, scenarios with signs, symptoms, and CVS examination findings were typed in the ChatGPT and the diagnosis was perfectly made for both, along with an appropriate management plan which was generally correct but cannot be totally dependent on in a clinical setting.

**Figure 9:**
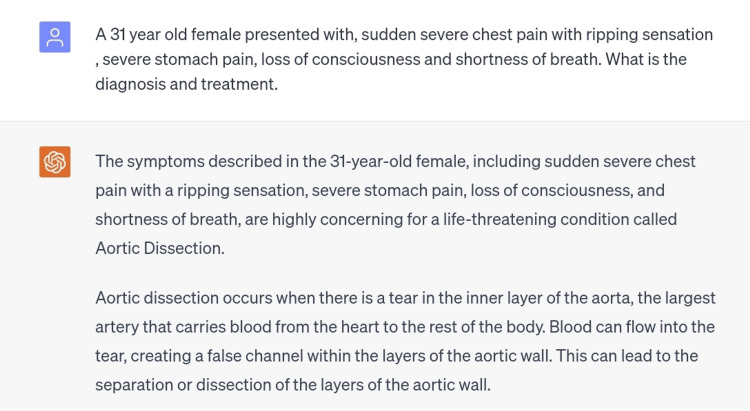
Scenario of aortic dissection.

The eighth scenario was of aortic dissection, a condition in which there is a tear in the aorta leading to dissection through the laminas and the formation of a new false lumen [10]. The patient in such a condition presents with low blood pressure, shortness of breath, chest pain, and dizziness. A scenario with similar signs and symptoms was inserted in ChatGPT and the diagnosis made was correct (Figure 9).

**Figure 10:**
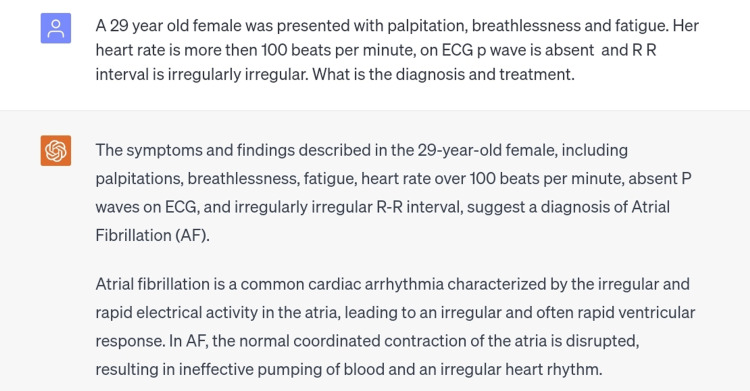
Scenario of atrial fibrillation.

The ninth scenario was atrial fibrillation, a condition in which abnormal electrical impulses start firing in the aorta, this condition is recognized by abnormal ECG findings such as heart rate higher than 100 beats per minute, absent P waves, and irregularly irregular rhythm [11]. A clinical picture consisting of the findings typical of this condition was typed in the chatbot and the diagnosis made was correct (Figure 10).

**Figure 11:**
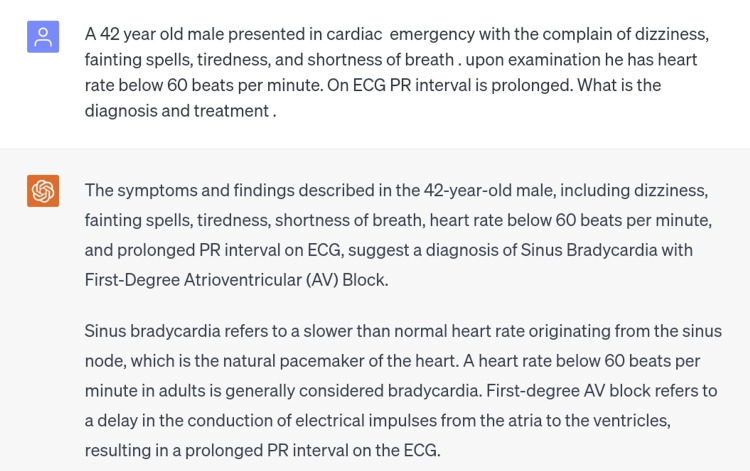
Scenario of first-degree atrioventricular block.

**Figure 12:**
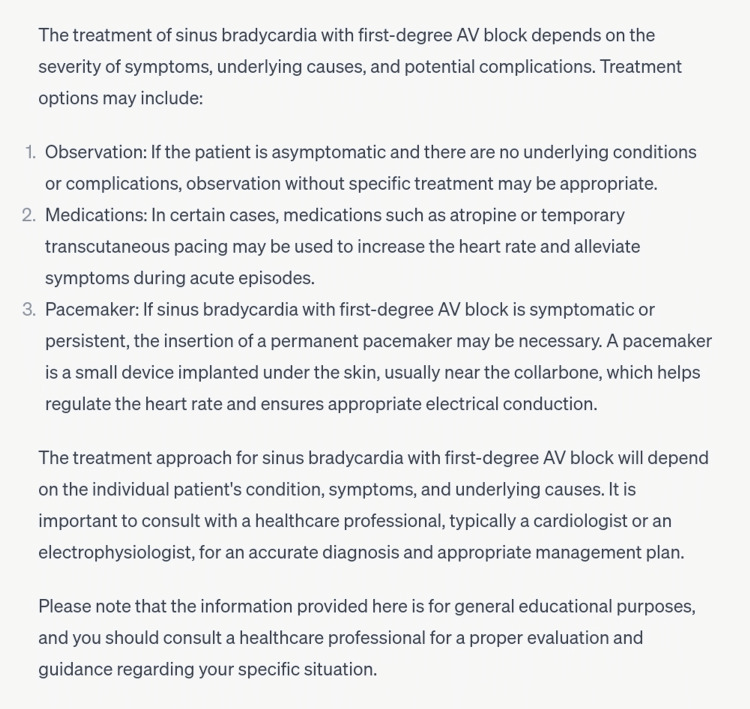
Management of first-degree atrioventricular block.

The 10th and final scenario was atrioventricular block. There are three degrees of AV block, which can be differentiated by their characteristic ECG findings [12]. The scenario inserted was of first-degree AV block and the diagnosis made was correct (Figure 11). In this case, the treatment plan was also correctly provided in an appropriate regimen (Figure 12).

The response provided by ChatGPT has effectively addressed the general management plan for the disorder, this does not only guide junior physicians while creating the treatment plan but also acts as a reassurance for the patients and their family while searching online about their condition. The response cannot act as a prescription or provide the exact information on what to use at a particular time or state of the patient or during an emergency situation. Out of the 10 clinical scenarios inserted in ChatGPT, eight were perfectly diagnosed, however, the other two answers given by ChatGPT were not entirely incorrect since those conditions were associated with the actual diagnosis.

Furthermore, the management plans and the treatment protocols that were given by ChatGPT were in line with the literature and current medical knowledge. The exact drug names and regimens were not provided but a general guideline that was given by this AI tool is definitely beneficial for junior doctors in getting an idea on how to proceed or refresh their previous knowledge.

## Discussion

This exploratory research delved into the capabilities that ChatGPT holds to understand and generate responses regarding clinical conditions in the healthcare domain in natural and understandable language, particularly in the field of cardiovascular disorders. The AI model has exhibited its proficiency in creating well-informed answers up to date with the research and guidelines related to the particular conditions. This AI model is easily accessible to the general population and the answers provided are in a simple and understandable language making it highly convenient to use.

The model presented consistent information based on its training data, mitigating the risk of human error or variability. With the use of ChatGPT, the need to dive into several websites and the confusion of deciding which website provides the correct information has become negligible, since it is providing complete, comprehensive information and also has an option of regenerating a response in case of dissatisfaction. This has reduced the amount of time and effort that it takes to find the correct information online. In cases when healthcare information is not sufficient, ChatGPT can provide a general guideline on management plan and treatment protocol [13].

However, it has its own limitations. It has been observed that in order to get the correct diagnosis the typical signs and symptoms as well as examination findings are needed. In the absence of any one of these, a provisional diagnosis is generated, though it is expected to have a list of differential diagnoses in case of any confusion or lack of information. Furthermore, the management and the treatment plans are general in terms that in every condition the treatment that you give depends upon the stage, state, and situation for instance the age, the comorbidities, and the stage and progression of the disease all in combination comprehend the medication needed.

Moreover, ethical concerns always emerge while relying on AI tools for medical advice as any misinterpretation can lead to detrimental outcomes. It has been observed that ChatGPT is an excellent tool to be used academically in writing articles, research papers, or in getting a deeper and more comprehensive insight into a particular medical condition. In clinical settings, it can definitely assist physicians in confirming their made diagnosis or in case of confusion making the correct diagnosis but in the presence of all typical signs, symptoms, and physical examination findings.

Limitations and future outcomes

In our study, we found a few limitations and potential future directions that could assist in further exploration and research. One of the limitations of this study which is necessary to address is that the clinical scenarios inserted had the typical picture, and unique and rare findings were not mentioned in it, this could be a challenge for AI tools such as ChatGPT to address. Furthermore, while asking for the management plan, we used the same scenario that was typed in for diagnosis, this gave a general management plan. In future studies, we would want to see the responses ChatGPT can give when the clinical scenarios are more complex with comorbidities and at different stages of the disease. If these limitations are addressed in future studies and worked on then the AI tools would definitely become a great dependable source for medical practitioners.

## Conclusions

To conclude, ChatGPT is a valuable resource in the field of medicine. Its comprehensive, and properly organized response in an understandable language has made it an effective and efficient tool to be used. Furthermore, it is easily accessible and free of cost through various digital platforms. However, it is crucial to note that its limitations such as the need for all associated and typical signs, symptoms, and physical examination findings and its inability to personalize treatments need to be acknowledged. Despite these challenges, ChatGPT has proved to be an efficient and effective tool both academically and in clinical setups.
